# Identification and Characterization of Atmospheric Nickel-Containing Particles in Guangzhou After the Implementation of the Clean Fuel Policy

**DOI:** 10.3390/toxics13050345

**Published:** 2025-04-26

**Authors:** Zaihua Wang, Xuanxiao Chen, Cheng Wu, Hong Ju, Zhong Fu, Xin Xiong, Ting Qiu, Yuchen Lu, Junjie He, Yaxi Liu, Haining Wu, Chunlei Cheng, Mei Li

**Affiliations:** 1 School of Energy Conservation and Safety, Guangdong Polytechnic of Environmental Protection Engineering, Guangzhou 510655, China; zaihuawang@163.com (Z.W.); 13727021686@163.com (X.C.); 19926137929@163.com (T.Q.); luyuchen620@163.com (Y.L.); 2 College of Environment and Climate, Institute of Mass Spectrometry and Atmospheric Environment, Guangdong Provincial Engineering Research Center for Online Source Apportionment System of Air Pollution, Jinan University, Guangzhou 510632, China; chengcl.vip@foxmail.com (C.C.); limei2007@163.com (M.L.); 3 Guangdong-Hongkong-Macau Joint Laboratory of Collaborative Innovation for Environmental Quality, Guangzhou 510632, China; 4 Guangzhou Sub-Branch of Guangdong Ecological and Environmental Monitoring Center, Guangzhou 510006, China; juh@gz.gov.cn (H.J.); hjj2225833@126.com (J.H.); 5 Key Laboratory of Organic Compound Pollution Control Engineering, School of Environmental and Chemical Engineering, Shanghai University, Shanghai 200444, China; 6 Hubei Academy of Eco-Environmental Sciences, Wuhan 430072, China; xx99227@163.com; 7 Department of Resource and Environment, Moutai Institute, Renhuai 564507, China; 17110740013@fudan.edu.cn; 8 Chishui River Middle Basin, Watershed Ecosystem, Observation and Research Station of Guizhou Province, Renhuai 564501, China; 9 Guangzhou Sub-Branch of Guangdong Maritime Safety Administration of the People’s Republic of China, Guangzhou 510260, China; wuhaining_gz@163.com

**Keywords:** nickel particle, single particle aerosol mass spectrometry, PM_2.5_, clean fuel policy, Guangzhou

## Abstract

Nickel, as a toxic trace element in fine particulate matter (PM_2.5_), has detrimental effects on both air quality and human health. Based on measurements from 2020 to 2021 using a single-particle aerosol mass spectrometer (SPAMS), this study investigates the properties of nickel-containing particles (NCPs) in Guangzhou. The composition, sources, and temporal trends of NCPs were evaluated and the impact of the clean ship fuel policy introduced in 2020 was also examined. The key findings include: (1) Nickel particles account for 0.08% number fraction of PM_2.5_, which is consistent with previously reported mass fraction in PM_2.5_. (2) Three distinct types of NCPs were identified, including Ni-fresh, Ni-aged, and Ni-ash. Each type exhibits unique characteristics in size distribution, wind direction dependence, sources, and temporal variations. Ni-fresh particles originate from shipping emissions in the Huangpu Port area 2 km away and are the major contributors to fine nickel particles in the region. (3) Ni-aged and Ni-ash particles, which carry secondary components, tend to be larger (>500 nm) and are representative of regional or background nickel particles. (4) The implementation of the clean ship fuel policy has effectively reduced the number concentrations of NCPs and is beneficial to regional and local air quality.

## 1. Introduction

Nickel (Ni) is a metal element that is widely distributed in the environment. In the atmosphere, nickel predominantly exists in particulate form, primarily originating from anthropogenic sources such as transportation, fossil fuel combustion, and metal smelting [1,2,3,4]. Additionally, natural processes, including windblown dust and volcanic eruptions, contribute to atmospheric nickel emissions. However, these naturally derived particles are generally larger, classified as coarse-mode particulates, and exhibit higher deposition velocities, resulting in a relatively short atmospheric residence time. In contrast, nickel emitted from combustion processes is more likely to be enriched in fine particulate matter (PM_2.5_) or ultrafine particles (PM_1_), remaining suspended in the atmosphere as aerosols for prolonged periods, thereby posing potential risks to both environmental quality and human health [5,6,7]. Fine particulate-bound nickel can penetrate deep into the pulmonary system, leading to respiratory damage and potentially contributing to adverse health effects such as inflammation, atherosclerosis, and oxidative stress [8,9] and increase cardiovascular mortality [10]. The health effects of nickel particulate matter on humans are greatly influenced by its chemical speciation and bioavailability. Soluble nickel compounds are more acutely toxic due to their bioavailability, leading to skin irritation and allergic contact dermatitis with dermal exposure [11]. In contrast, insoluble nickel species, such as sulfidic (Ni_3_S_2_) and oxidic (NiO) forms, are linked to chronic respiratory pathologies [12]. Epidemiological studies highlight that occupational inhalation of these less-soluble nickel particles in refinery dust elevates the risk of lung and nasal cancers, with sulfidic nickel demonstrating the highest carcinogenic potential [13,14]. The International Agency for Research on Cancer (IARC) classifies nickel compounds as Group 1 carcinogens, while metallic nickel is categorized as Group 2B [11,14]. Mechanistically, nickel-induced carcinogenesis involves oxidative stress, DNA damage, and epigenetic alterations [11]. Soluble Ni^2+^ ions can penetrate cellular membranes, generating reactive oxygen species (ROS) that oxidize lipids, proteins, and DNA, leading to genomic instability [15]. Insoluble particles, though less bioavailable, persist in lung tissues, inducing chronic inflammation and macrophage-driven phagocytosis [16]. Additionally, nickel interferes with histone acetylation and DNA methylation, silencing tumor suppressor genes and promoting malignant transformation [11].

To investigate the environmental and health effects of atmospheric nickel, it is essential to monitor its concentration. Nickel in the atmosphere typically exists in trace amounts, with mass concentrations generally in the range of a few nanograms per cubic meter [2,5,6]. Traditional offline analytical methods, while capable of measuring nickel concentrations, suffer from low temporal resolution, as trace-level substances require prolonged sampling durations to accumulate sufficient material on filter membranes for analysis. Consequently, these methods provide only average concentration values and fail to capture critical information regarding the specific physicochemical state of nickel in atmospheric particles, such as the types of particles it adheres to and their size distribution. These details necessitate complementary analytical techniques. Single-particle aerosol mass spectrometry (SPAMS) has emerged as a powerful tool capable of providing such crucial information. By analyzing individual aerosol particles in real-time, SPAMS offers insights into the mixing state, size distribution, and number concentration of nickel-containing particles (NCPs), making it an ideal method for characterizing particulate-phase metals [17,18,19].

The combustion of heavy fuel oil (HFO) in marine vessels is a significant source of atmospheric nickel, particularly in coastal and port areas. Previous studies have used single-particle mass spectrometry (SPAMS) to analyze ambient particulate matter in port regions, specifically focusing on real-time monitoring of freshly emitted ship exhaust particles near docks. Unlike other techniques, such as online X-ray fluorescence (XRF), which can only measure the bulk concentration of nickel, SPAMS allows for the characterization of the mixing state and size of individual nickel-containing particles (NCPs). This capability is valuable for identifying the source of these particles at the single-particle level. These emissions form a series of distinct particle concentration peaks, within which characteristic ship-derived particles can be identified. Their mass spectra exhibit strong signals for vanadium, nickel, iron, sodium, elemental carbon (EC), and sulfates [17,20]. Similar studies have been conducted domestically in the Shanghai port area, where SPAMS monitoring revealed that variations in vanadium–nickel particle concentrations were synchronized with peak levels of gaseous pollutants such as SO_2_ and NO_x_. Additionally, concentration–wind direction analysis further confirmed ship emissions as the source [19]. Beyond ship emissions, SPAMS has also been employed to monitor particulate matter around steel plants. Research has identified a specific type of nickel-containing particle characterized by a dominant nickel signal in the mass spectrum. In the negative ion mode, these particles exhibit strong nitrate signals but nearly no sulfate signals, suggesting a close association with steel rolling processes [1].

The Sulfur Emission Control Areas (SECAs) policy was first introduced in 2005 by the International Maritime Organization (IMO) for the Baltic Sea and North Sea to mitigate sulfur oxide impacts on sensitive ecosystems. The North American SECA (including U.S. and Canadian waters) was established in 2010 with a sulfur cap of 1.0%, followed by the U.S. Caribbean SECA in 2012. With the implementation of stricter ship emission control measures, China has enforced nationwide clean fuel policies within SECAs, including both coastal and inland control zones. As of January 1, 2020, the sulfur content of all marine fuels used within SECAs must not exceed 0.5% m/m, while for ocean-going vessels entering inland control zones, the sulfur limit is further restricted to 0.1% m/m. It is found that the clean fuel policy led to significantly lowered emissions of metals such as nickel and vanadium in Shanghai [21]. Previous studies in the Pearl River Delta (PRD) region mainly focus on vanadium [22] and volatile organic compounds (VOCs) [23]. How the implementation of SECAs in the PRD affects the atmospheric nickel particles remains unknown and requires investigation. Consequently, continuous monitoring is essential to track changes in shipborne particulate emissions following the implementation of these fuel policies. In 2024, Guangzhou Port ranked sixth in the world for container throughput and is the largest coastal and maritime transport hub in southern China. This study utilizes monitoring data from a SPAMS stationed near the Huangpu Port of Guangzhou, covering two full years (2020–2021), which provides a unique opportunity to perform a post-policy air quality evaluation. By analyzing the composition and size distribution of nickel-containing particles, the effectiveness of the Clean Fuel Policy can be examined.

## 2. Measurement Methods

### 2.1. The Measurement Site

**Figure 1:**
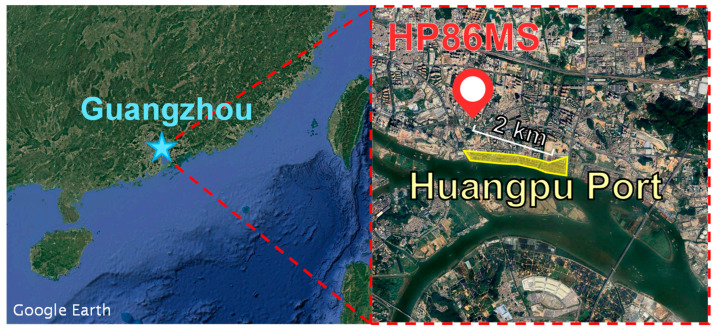
The satellite map of Guangzhou and the location of the sampling site.

The sampling site was located at the Guangzhou Huangpu No. 86 Middle School (HP86MS), approximately 2 km from Huangpu Port (Figure 1). Huangpu Port, situated on the northern bank of the Pearl River in Guangzhou, is the largest coastal and maritime transport hub in southern China. The monitoring site is surrounded by dense traffic networks, industrial zones, and residential areas, making it subject to significant influences from industrial, vehicular, and domestic emissions. This location offers a representative setting for studying Guangzhou’s complex atmospheric environment. The monitoring period spanned from 1 January 2020 to 31 December 2021, covering two full years. In addition to single-particle aerosol monitoring, the dataset included meteorological parameters such as temperature, relative humidity, wind speed, and wind direction, as well as hourly concentrations of PM_10_, PM_2.5_, and gaseous pollutants. All monitoring instruments were regularly calibrated following national standards to ensure data accuracy and reliability.

### 2.2. Monitoring Instruments

To ensure high-resolution and real-time monitoring of atmospheric particles, a Single-Particle Aerosol Mass Spectrometer (SPAMS-0515, Hexin Analytical Instrument Co., Ltd., Guangzhou, China) was deployed at the monitoring site. The SPAMS-0515 enables real-time characterization of individual aerosol particles, providing critical information on their chemical composition, size distribution, and mixing state. The working principle of the SPAMS-0515 is briefly described as follows: Atmospheric particulate matter is first drawn into the instrument through a sampling inlet and then passes through a micro-orifice (inner diameter: 0.1 mm) before entering the aerodynamic focusing lens (AFL). The AFL constrains and accelerates the particle trajectories, establishing a functional relationship between particle velocity and aerodynamic diameter. After leaving the AFL, particles pass sequentially through two laser beams set at a fixed distance apart, producing two scattering signals. The time difference between the two scattering signals is used to calculate the particle’s velocity in vacuum. Then, the diameter can be derived using a predefined velocity–size relationship obtained from standard polystyrene latex sphere calibration. Once the particles leave the sizing region, they enter the mass spectrometry ionization zone, where a pulsed laser (wavelength: 266 nm) is triggered at an optimal time to ionize the particles. The resulting positive and negative ion spectra are recorded by a dual-polarity time-of-flight mass spectrometer (TOF-MS), enabling the determination of both particle size and chemical composition [24]. To ensure data accuracy and instrument stability, routine maintenance procedures were implemented, including: checking the SPAMS inlet pressure to maintain optimal particle flow; calibrating the mass spectrum axis to ensure accurate ion identification; and monitoring the ionization laser energy to ensure consistent particle ionization efficiency. These quality control measures ensured the reliability of the SPAMS data throughout the monitoring period.

### 2.3. Data Processing

In this study, the dataset from 2020 to 2021 included approximately 310 million recorded particle size measurements and 40 million individual particle ion mass spectra (positive and negative). To efficiently manage this extensive dataset, an organized framework was implemented to facilitate rapid retrieval, visualization, classification, and statistical analysis. The classification of SPAMS particles was conducted using two complementary methods: feature-based peak search and cluster analysis based on spectral similarity. The first method involved identifying particles with characteristic mass spectral peaks, enabling a rapid but dimensionally limited search. The second method employed clustering algorithms to group particles with similar mass spectra, allowing for a more comprehensive classification. To accurately identify nickel-containing particles, a combination of these approaches was applied. Initially, all particles exhibiting potential nickel ion peaks (identified using mass-to-charge ratio, *m*/*z* = +58 and its primary isotope *m*/*z* = +60) were extracted. However, as these mass-to-charge ratios could also correspond to non-nickel species, additional filtering was required. Subsequently, clustering algorithms were used to group the extracted particles based on spectral similarity. Clusters that did not exhibit the expected nickel spectral characteristics were removed, and the remaining clusters were merged to form the final dataset of nickel-containing particles. This hybrid approach minimized interference from non-nickel species, improving the accuracy of classification and ensuring reliable results for further source apportionment and trend analysis. In order to ensure the consistency of the classification criteria, all Ni-containing single particles measured over two years were included in a whole dataset. The Ni-containing particles were classified into three types including Ni-fresh, Ni-aged, and Ni-ash according to the presence of elemental carbon clusters (EC, C_n_^+/−^), nitrate, and mineral dust and fly ash compositions (Al^+^, Cu^+^, Cr^+^, Li^+^, Pb^+^, and SiO_3_^−^).

## 3. Results and Discussion

### 3.1. Identification of Nickel-Containing Particles from Interfering Ions

**Figure 2:**
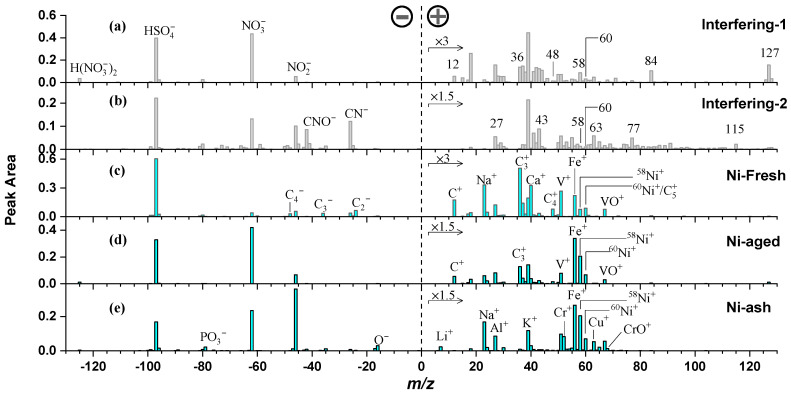
Positive and negative mass spectra of two interfering particle types (**a**,**b**) and nickel particles (**c**–**e**).

To retrieve nickel-containing particles, *m*/*z* = +58 and +60 were used as the defining criteria. This relatively broad selection approach aimed to minimize the risk of missing any potential nickel-containing particles. Based on this criterion, approximately 696,000 particles were identified, accounting for 1.7% of the total detected particles (40.65 million). However, due to mass spectral interferences at *m*/*z* = +58 and +60, the initial search results included non-nickel-containing particles (interfering particles). To eliminate these interferences, all potential nickel-containing particles were further clustered using an algorithmic approach. Given their compositional differences, true nickel-containing particles and interfering particles formed distinct clusters. By analyzing the mass spectral characteristics, temporal trends, and size distributions of each cluster, interfering particles were systematically identified and removed. Two representative types of interfering particles (interference-1 and interference-2) were identified, and their average mass spectra are shown in Figure 2a,b. These spectral features helped distinguish authentic nickel-containing particles from those influenced by other species, ensuring a high-confidence dataset for subsequent analysis.

From the mass spectral characteristics, the interference-1 particle exhibited +58 and +60 peaks along with several other prominent ion peaks, particularly +84 and +127. Additionally, statistical analysis revealed a strong positive correlation between the intensity of the +58 peak and the +84 and +127 peaks, suggesting that +58 may be a fragment ion associated with +84 and +127 rather than a nickel-related ion. Further spectral analysis showed that particles containing +84 and +127 peaks generally exhibited strong +58 signals, whereas those lacking these peaks showed little to no +58 intensity. Since +84 and +127 do not correspond to the known nickel-related ion NiO^+^ (*m*/*z* = 74), it is likely that the +58 peak originates from organic fragment ions rather than Ni^+^. Previous studies have identified *m*/*z* = +58 (C_3_H_8_N^+^) as a characteristic peak of organic amines [25]. Organic amines are highly volatile compounds that can react with atmospheric acidic gases to form salts and subsequently integrate into existing particles. Further examination of the +84 and +127 peaks revealed their presence across a wide range of particle types, including mineral dust, fly ash, sea salt, and EC, suggesting that these ions may originate from homogeneous atmospheric reactions rather than specific emission sources. Moreover, additional spectral features, such as ^12^C^+^, ^36^C_3_^+^, and ^48^C_4_^+^, indicate that the +60 peak is more likely associated with ^60^C_5_^+^ rather than ^60^Ni^+^. From statistical data, interference-1 particles were the dominant type of interfering particles, with their occurrence peaking between January and March 2020. This temporal pattern suggests that the presence of these interfering particles may be linked to specific seasonal atmospheric processes or episodic emissions.

The interfering-2 particles exhibited a mass spectrum rich in organic fragment ions (Figure 2b), characterized by a continuous peak distribution in the low mass-to-charge (*m*/*z*) region (1–100 amu). This pattern is a typical feature of atmospheric organic matter (OM) particles [26,27]. These particles exhibit prominent organic fragment peaks including +27 (C_2_H_3_^+^), +43 (C_2_H_3_O^+^, a marker for oxidized organic species), and +77 (C_6_H_5_^+^, indicative of benzene-related compounds), as well as +115, which is associated with aromatic compounds. The presence of these peaks suggests that these particles contain aromatic hydrocarbons and have undergone atmospheric oxidation processes. Due to the abundance of organic fragments in their spectra, these particles frequently exhibited +58 and +60 peaks, leading to potential misclassification as nickel-containing particles. However, a comparative analysis of their mass spectral features and temporal concentration trends revealed significant differences between organic-rich particles and true nickel-containing particles, making their distinction relatively straightforward.

**Figure 3:**
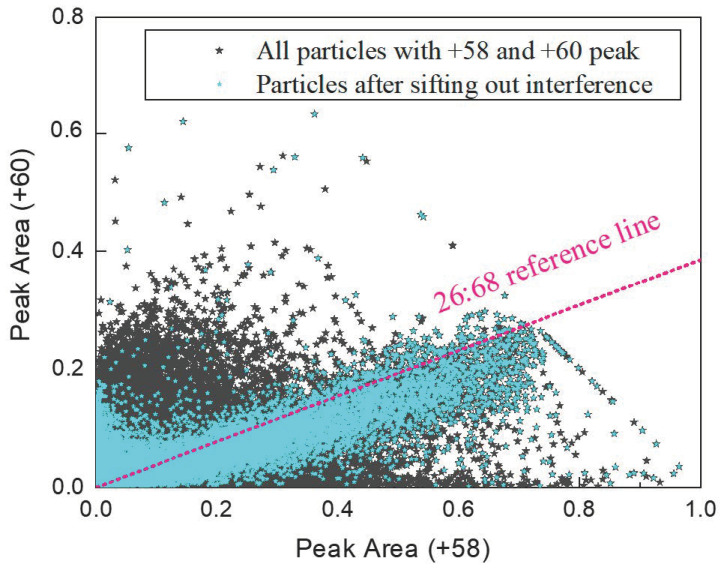
Scatter plot of peak area at *m*/*z* +58 and +60 of individual nickel particles. The markers in light blue represent authentic NPCs while the markers in black refer to interfering ions.

After eliminating interfering particles, the genuine nickel-containing particles amounted to around 32,000, representing 0.08% of the total detected particles. This proportion aligns well with the mass concentration-based estimates of nickel in atmospheric PM_2.5_ (0–0.15%) reported in previous studies [2,5,6,28]. Following the removal of interference, a significant change was observed in the peak area distribution at *m*/*z* = +58 and +60 in the nickel particle mass spectra. The natural isotope abundance of ^58^Ni and ^60^Ni are 68% and 26% of total nickel, respectively [29,30]. After interference removal, the peak area ratio of ^58^Ni^+^ to ^60^Ni^+^ closely matched this expected natural abundance ratio of 26:68 (Figure 3). This consistency validates the accuracy of the refined dataset, confirming that the remaining particles more reliably represent true nickel-containing aerosols.

### 3.2. Composition and Size Distribution of Nickel-Containing Particles

**Figure 4:**
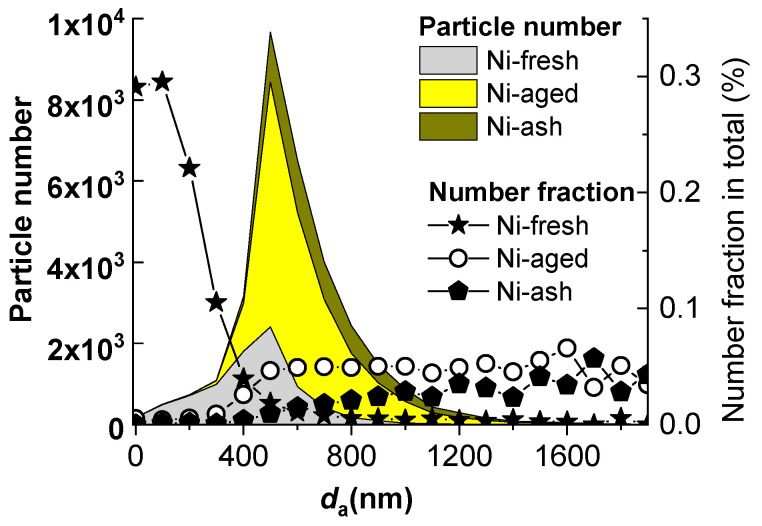
Particle number distribution of three nickel particle types (shown in filled areas) and their number fractions (shown by lines with markers).

Based on mass spectral characteristics, nickel-containing particles were classified into three categories: freshly emitted nickel particles (Ni-fresh), aged nickel particles (Ni-aged), and fly ash-type nickel particles (Ni-ash). Their average mass spectral features and size distributions are shown in Figure 2c–e and Figure 4. Ni-fresh particles primarily consist of EC (C_n_^+/−^, n = 1, 2, 3, … series ions), sulfates (HSO_4_^−^), and metallic elements such as vanadium (V^+^, VO^+^), nickel (Ni^+^), iron (Fe^+^), sodium (Na^+^), and calcium (Ca^+^). Notably, their negative ion mass spectra exhibit little to no nitrate signals (NO_2_^−^, NO_3_^−^), distinguishing them from aged or secondary particles. Morphologically, freshly emitted EC is primarily present as carbonaceous microspheres with a branched, fluffy structure, contributing to their small aerodynamic diameter and enhancing their role in the ultrafine particle mode. The size distribution of Ni-fresh particles is predominantly concentrated below 400 nm, with their number fraction increasing significantly as particle size decreases (Figure 4). This pattern strongly indicates that Ni-fresh particles are closely associated with combustion sources, particularly ship emissions, where nickel is typically released in ultrafine forms.

Ni-aged particles exhibit positive ion mass spectral features similar to Ni-fresh particles, but they are distinguished by the presence of prominent nitrate signals in their negative ion spectra (Figure 2d). Ni-aged particles are the result of atmospheric aging of Ni-fresh particles, where secondary processes lead to the accumulation of nitrates, sulfates, and other secondary species, resulting in a notable increase in particle size. Their size distribution is predominantly above 400 nm, with the number fraction stabilizing at approximately 0.05% in the >500 nm range. Previous single-particle studies have identified Ni-fresh and Ni-aged particles as common types of ship-emitted aerosols, and the mass spectral features and size distributions obtained in this study are highly consistent with those reported in the literature [17,19,31].

Ni-ash particles are primarily distinguished from Ni-fresh and Ni-aged particles by the absence of carbon cluster ions (C_n_^+/−^). Additionally, their positive ion mass spectra exhibit characteristic mineral or fly ash elements, including Al^+^, Cu^+^, Cr^+^, Li^+^, and Pb^+^, while their negative ion spectra show distinctive O^−^, OH^−^, PO_3_^−^, and SiO_3_^−^. These spectral characteristics indicate that Ni-ash particles are predominantly composed of mineral dust or fly ash, without EC content [1,18,20]. Ni-ash particles are typically larger (>500 nm), and their number fraction increases with particle size, making them the largest NCPs in particle size among the three categories.

### 3.3. Source Analysis of Nickel-Containing Particles

**Figure 5:**
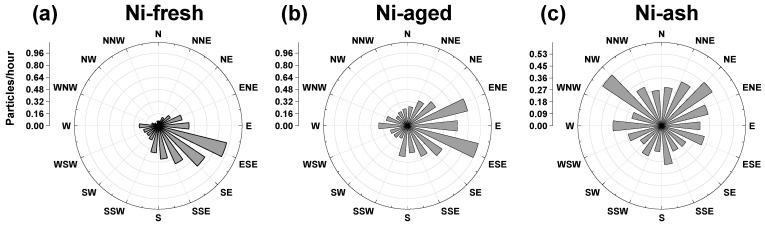
Wind roses of three nickel particle types. The radius of the grey sector represents the frequency of particles coming from the corresponding direction.

Analysis of particle properties revealed significant differences among the three types of nickel-containing particles, reflecting their distinct sources or influencing factors. SPAMS enables the differentiation of individual particles, allowing for more precise source attribution of nickel-containing aerosols. By combining nickel particle concentrations with wind direction observations, the frequency of different particle types under various wind directions was examined, as illustrated in the wind rose diagram (Figure 5). Vanadium (V) and nickel (Ni) are key tracer metals for HFO combustion emissions. As shown in Figure 5, Ni-fresh particles exhibit a strong wind direction dependency, with their highest concentrations observed under southeasterly winds, while concentrations from other directions decline rapidly. Notably, concentrations from the west to northwest (inland) directions approach zero, indicating that Ni-fresh particles are strongly influenced by localized sources [17,19]. Huangpu Port is located southeast of the monitoring site, approximately 2 km away (Figure 1). Given this spatial alignment, ship emissions in and around the port are likely the primary source of Ni-fresh particles. The wind rose analysis from both 2020 and 2021 consistently exhibited this directional dependence, suggesting that this source pattern remains stable.

As shown in Figure 5b,c, Ni-aged and Ni-ash particles exhibit lower wind direction dependence than Ni-fresh particles, with a more widespread distribution across different wind directions but with higher concentrations in specific sectors. Ni-aged particles originate from the atmospheric aging of Ni-fresh particles, meaning their primary source remains HFO combustion. Since Ni-aged particles have a longer atmospheric residence time than Ni-fresh particles, they are likely to disperse further from their emission sources, making them regionally transported HFO combustion aerosols. The wind direction analysis indicates that Ni-aged concentrations are higher under easterly winds, suggesting that their main sources are ship emissions from the eastern or southeastern marine areas. In contrast, Ni-ash particles differ from Ni-aged particles in their wind direction dependence. Higher Ni-ash concentrations were observed under northern inland winds, whereas concentrations were lower under southerly winds from the ocean, indicating that Ni-ash is predominantly associated with land-based emissions. Previous studies have shown that filterable particulate matter (FPM) emitted from coal-fired power plants and waste incinerators contains both nickel and vanadium [32]. Additionally, potential Ni-ash emission sources include steel production, metal smelting, and other industrial processes [1,2,18,20].

### 3.4. Temporal Trends of Nickel-Containing Particles

**Figure 6:**
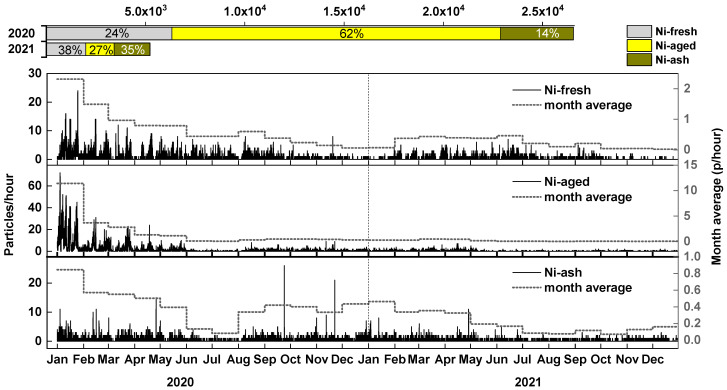
Particle number trends of three nickel particle types (left axis) and their monthly average trends (right axis). The two bars above indicate nickel particle numbers and respective percentages of the three types in 2020 and 2021.

Previous analyses have identified ship emissions from HFO combustion as the primary source of atmospheric nickel, particularly in the form of Ni-fresh and Ni-aged particles. The implementation of China’s clean fuel policy on 1 January 2020, which mandated a reduction in sulfur content in marine fuels, was expected to have a significant impact on atmospheric nickel concentrations [21]. To assess this impact, we analyzed the hourly and monthly average number concentrations of nickel-containing particles from 2020 to 2021. Figure 6 illustrates the temporal trends in nickel particle concentrations, along with the absolute particle counts and relative proportions of the three nickel particle types in 2020 and 2021. The results indicate a sharp decline in Ni-fresh and Ni-aged particle concentrations immediately after the policy took effect. A sharp reduction was found in early 2020, and the level was stable from April 2021 to the end of 2021. Based on annual averages, the concentration of Ni-fresh particles in 2021 decreased by 68.6% compared to 2020, while Ni-aged particles declined by 91.4%. These findings demonstrate the effectiveness of the clean fuel policy in reducing atmospheric nickel concentrations.

In 2021, the concentration of Ni-ash particles decreased by 50.1% compared to 2020, a significantly smaller reduction than that observed for Ni-fresh (68.6%) and Ni-aged (91.4%) particles. This discrepancy likely stems from the different primary sources of Ni-ash compared to the other two particle types. As previously analyzed, Ni-ash is mainly derived from land-based emission sources, meaning that the direct impact of clean fuel policy on shipping emissions is limited. The observed decrease in Ni-ash particles may instead be attributed to reductions in industrial activity during the COVID-19 pandemic in 2020, which led to lower emissions from industrial processes, coal combustion and metal smelting. The differences in reduction rates among Ni-fresh, Ni-aged, and Ni-ash also led to a shift in the relative contribution of nickel emission sources. In 2020, atmospheric nickel was predominantly sourced from ship HFO combustion, accounting for 86%, while land-based sources (Ni-ash) contributed only 14%. However, following the implementation of the clean fuel policy, the proportion of Ni-ash particles increased to 35% in 2021, indicating that land-based sources became a more significant contributor to atmospheric nickel.

**Figure 7:**
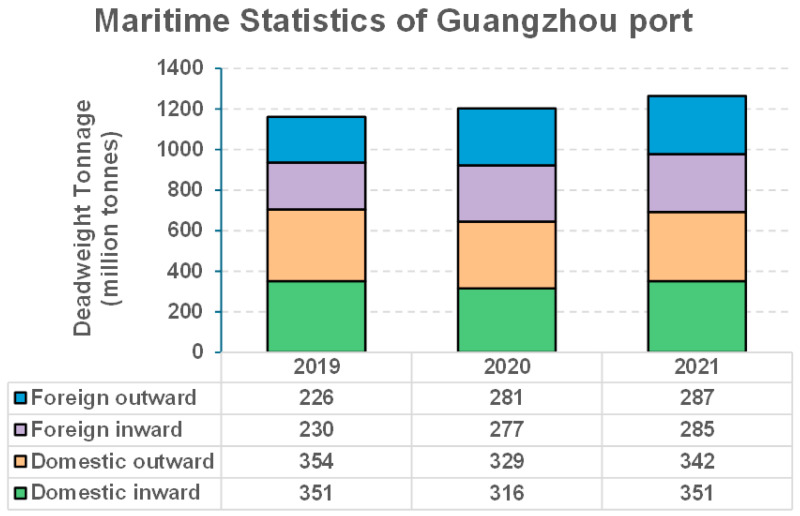
Maritime statistics of inward and outward ship traffic at Guangzhou port by million deadweight tonnage (DWT) from 2019 to 2021.

The COVID-19 pandemic had a significant impact on economic activities in 2020 and 2021, prompting an examination of the maritime statistics for Guangzhou Port from 2019 to 2022. Guangzhou Port comprises three areas: Huangpu, Xinsha, and Nansha. As illustrated in Figure 7, the total annual inward and outward traffic by deadweight tonnage (DWT) experienced steady growth during this period, increasing from 1161 million tons in 2019 to 1265 million tons in 2021. Therefore, unlike the suppressed economic activities in other sectors (e.g., ground transport, aviation), shipping is not affected at Guangzhou Port as steady growth remains during the pandemic. Previous long-term studies have shown that meteorology contributions are usually small (<20%) for the observed reduction in air pollutants in the PRD and other regions of China [33,34]. Therefore, it can be concluded that the reduction in nickel seen from 2020 to 2021 was primarily due to the clean fuel policy, as shipping activity has experienced steady growth.

Previous studies around the world have shown the effectiveness of SECAs on air pollution mitigation near the port area [35]. A study at two Canadian cities on the Atlantic and Pacific coast over the 2010–2016 period showed that SECA low-sulfur regulations reduced residual oil PM_2.5_ by 94–95% (0.24–0.25 μg/m^3^) and anthropogenic sulfate PM_2.5_ by 47–58% (0.71–0.78 μg/m^3^) [36]. A study at the largest grain port in Latin America revealed a significant decrease in PM_2.5_ nickel from 5.8 to 2.2 ng/m^3^ [37]. A study in New York State from 2005 to 2019 demonstrated that CO, NO_2_, SO_2_, and PM_2.5_ were effectively reduced by the combination of SECAs and other regulatory measures [38]. Consistent with previous studies that indicate a reduction in sulfur content, this study demonstrates that the levels of toxic heavy metals, such as nickel, can also be decreased. Maritime transport is an important source of air pollution and health risk factors, globally causing ∼265,000 premature deaths in 2020 [39]. This study, along with previous studies around the world, has proven that the SECA Clean Fuel sulfur cap regulation benefits not only the reduction in routine air pollutants like SO_2_ but is also effective in lowering toxic air pollutants like nickel and vanadium, which can promote chronic inflammation and malignant transformation, leading to elevated risk of lung and nasal cancers. That would be very helpful in protecting public health in terms of mortality and morbidity.

### 3.5. Reduction in Vanadium-Associated Nickel Particles

**Figure 8:**
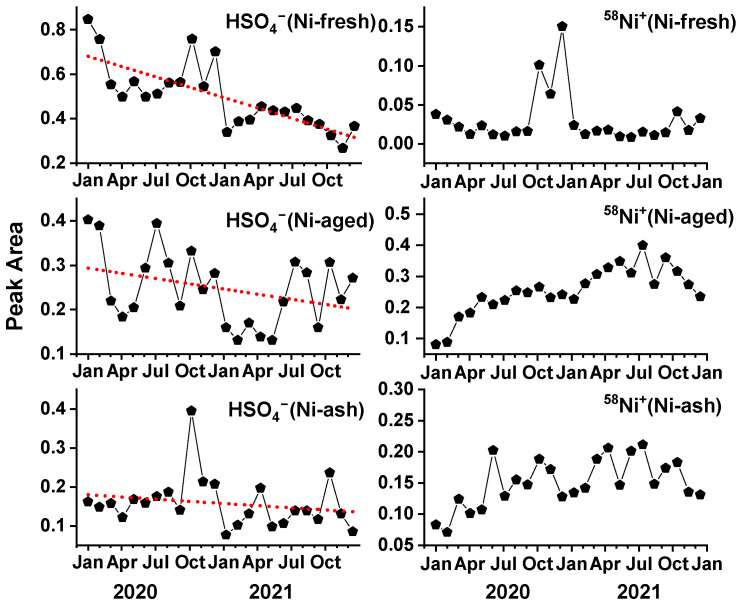
Mass spectra peak intensity (sulfate: ^97^HSO_4_^−^, nickel: ^58^Ni^+^) variations of three nickel particle types during the 2-year sampling period. The data points represent the monthly average peak area of nickel particles. Red dotted lines are linear fits of monthly data points.

To track the impact of the clean fuel policy on particle composition, the changes in mass spectral peak signals of nickel-containing particles were analyzed. The transition to low-sulfur fuel has significantly reduced SO_2_ emissions from ships, which in turn influenced the sulfate (HSO_4_^−^) signal intensity in ship-derived nickel particles. Figure 8 (left panel) illustrates the monthly mean sulfate signal intensity for the three types of nickel-containing particles over 24 months. The results indicate that Ni-fresh particles exhibited the most pronounced decline in sulfate signals, with the average peak area decreasing from 0.7 at the beginning of the policy implementation to 0.38 by December 2021. Since Ni-fresh represents freshly emitted particles, this trend directly reflects the impact of the clean fuel policy, demonstrating a significant reduction in sulfate content in ship emissions. In contrast, Ni-aged particles showed a smaller reduction in sulfate signals. This is likely due to the formation of secondary sulfates during atmospheric aging processes, which partially offset the initial reduction in primary sulfate emissions, as shown in Figure 8. Meanwhile, Ni-ash particles did not exhibit a clear trend in sulfate signal variation. This is likely attributed to their different emission sources. The clean fuel policy primarily targeted ship emissions, whereas Ni-ash is predominantly derived from land-based sources such as coal combustion and steel manufacturing. As a result, sulfate in Ni-ash particles remained relatively stable throughout the monitoring period.

The changes in positive ion mass spectral signals of nickel-containing particles were more complex, as illustrated by the ^58^Ni^+^ trend shown in the right panel of Figure 8. The nickel signal in Ni-fresh particles remained relatively stable throughout the monitoring period, except for an increase observed during October–December each year, the specific cause of which remains unclear. In contrast, the nickel signal intensity in Ni-aged particles exhibited an increasing trend. Here, the signal intensity was represented by the relative peak area, defined as the ratio of a specific peak area to the total mass spectral peak area. Consequently, an increase in relative peak area may indicate either an actual increase in nickel content or a reduction in the peak areas of other chemical species. Mass spectral data showed that organic peak areas were higher on average during January-March 2020 compared to other periods, suggesting that the observed increase in Ni signal may be partly attributed to a decrease in organic peak intensity rather than a direct increase in nickel concentration.

The above discussion indicates that the implementation of the clean fuel policy led to changes in the mass spectral characteristics of nickel-containing particles, reflecting compositional differences associated with different fuel types. Based on the trend in nickel-containing particle number concentrations, the extent of compositional changes was smaller than the reduction in particle number concentrations, suggesting that the primary effect of the fuel policy was the overall reduction in the number concentration of nickel-containing particles rather than a fundamental alteration of their composition. In future studies aimed at source apportionment of HFO combustion particles, it will be necessary to adjust source profiles accordingly to more accurately reflect updates in ship fuel regulations and their impact on atmospheric particulate matter composition.

## 4. Conclusions

Based on two full-year single-particle monitoring data, this study systematically analyzed the properties, sources, and concentration trends of nickel-containing particles in Guangzhou. The impact of the 2020 ship clean fuel policy on atmospheric nickel was assessed. The key findings are as follows:

(1) Nickel-containing particles accounted for a relatively small proportion of total atmospheric particles, with an average number fraction of 0.08%, which is consistent with the mass fraction in previous studies;
(2) Nickel-containing particles exhibited distinct chemical compositions and were classified into three types: freshly emitted HFO combustion particles, aged HFO combustion particles, and mineral dust/fly ash-type particles. Among these, freshly emitted HFO combustion particles were enriched in EC, sulfates, and characteristic metallic components, making them a major contributor to ultrafine nickel-containing particles near the port areas;
(3) Aged HFO combustion particles and mineral dust/fly ash-type particles were predominantly distributed in the accumulation mode (>500 nm) and were enriched in secondary components, representing regional or background nickel-containing particles. Mineral dust/fly ash-type particles primarily originated from land-based emissions, whereas aged HFO combustion particles were mainly derived from regional ship emissions;
(4) The implementation of the ship clean fuel policy greatly reduced the concentration of nickel-containing particles, leading to significant improvements in both local and regional air quality. Additionally, the policy led to changes in nickel particle composition and altered the relative contribution of different nickel emission sources. The success of the SECA policy has demonstrated that coordinated air quality management measures are effective in addressing regional air pollution, and this model can be applied not only to the shipping industry but also to other sectors.

## Data Availability

The data presented in this study are available on request from the first author due to legal restrictions. The SPAMS data are owned by a government agency and their disclosure requires a case-by-case application. Requests for data should be addressed to Zaihua Wang (zaihuawang@163.com).
